# Supplementary material for: Extant Earthly Microbial Mats and Microbialites as Models for Exploration of Life in Extraterrestrial Mat Worlds
Source: Life (Basel). 2021 Aug 27;11(9):883. doi: 10.3390/life11090883 (PMC8468739; doi:10.3390/life11090883)
Supplement: Supplementary file 1 [file life-11-00883-s001.zip › life-1301691-supplementary.pdf]

life

MDPI

SUPPLEMENTAL INFORMATION

Extant earthly microbial mats and microbialites as models for exploration of life in extraterrestrial mat worlds

Bopaiah Biddanda [1,*], Anthony Weinke [1], Ian Stone [1], Scott Kendall [2], Phil Hartmeyer [3], Wayne Lusardi [3], Stephanie Gandulla [3], John Bright [3] and Steven Ruberg [4]

[1] Annis Water Resources Institute, Grand Valley State University, Muskegon, MI 49441, USA; weinkea@mail.gvsu.edu (A.W.); stoneia@mail.gvsu.edu (I.S.)
[2] Biology Department, Muskegon Community College, Muskegon, MI 49442, USA; scott.kendall@muskegoncc.edu
[3] Thunder Bay National Marine Sanctuary, Office of National Marine Sanctuaries, National Oceanic and Atmospheric Administration, Alpena, MI 49707, USA; phil.hartmeyer@noaa.gov (P.H.); wayne.lusardi@noaa.gov (W.L.); steph.gandulla@noaa.gov (S.G.); john.bright@noaa.gov (J.B.)
[4] Great Lakes Environmental Research Laboratory, National Oceanic and Atmospheric Administration, Ann Arbor, MI 48108, USA; steve.ruberg@noaa.gov
* Correspondence: biddandb@gvsu.edu; Tel.: (1 616 331 3978)

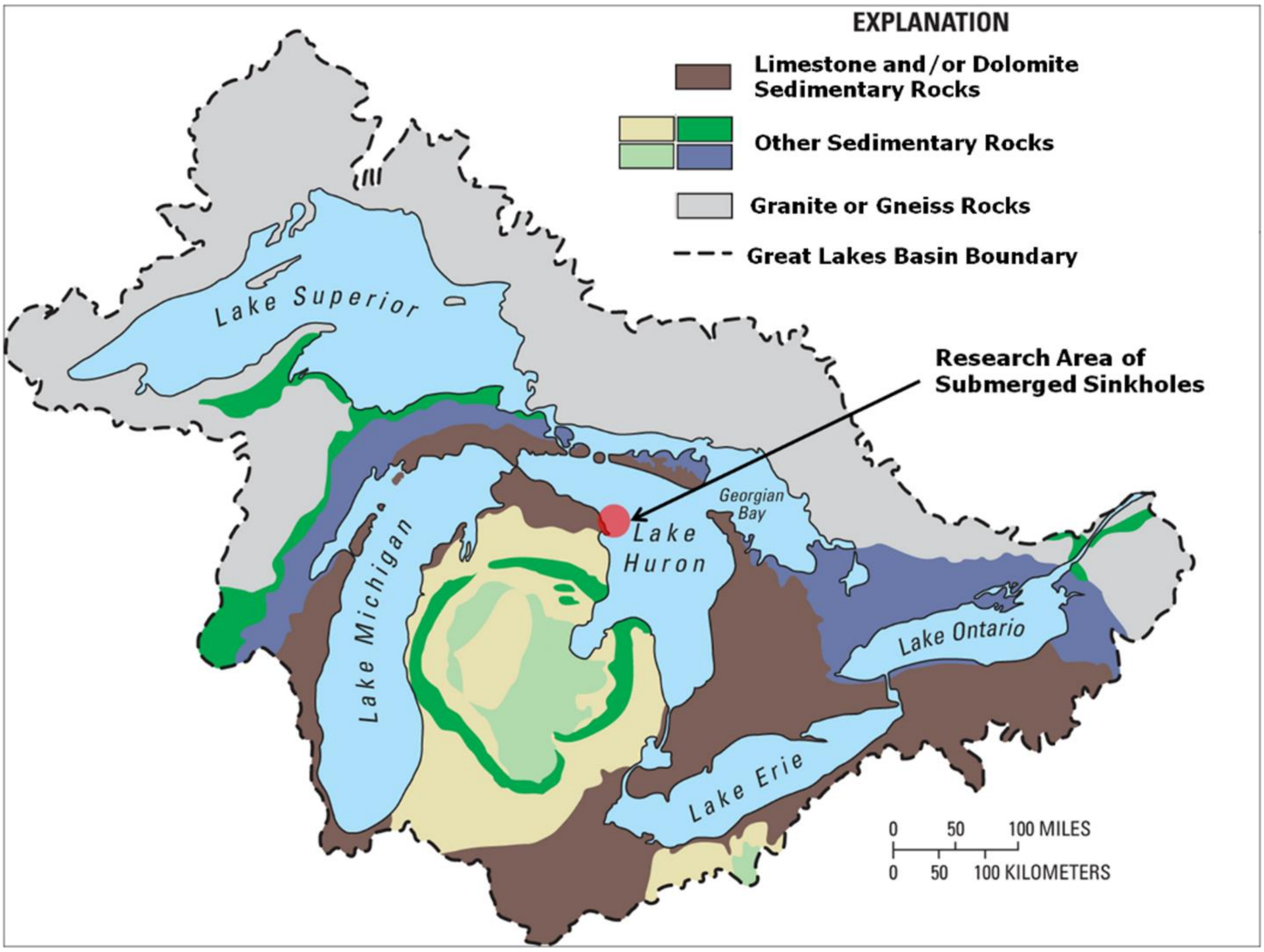

Figure S1. A map of the North American Great Lakes basin showing the geological context and the study site in Lake Huron. The Great Lakes basin: Surface geology map of the North American Laurentian Great Lakes basin showing abundance of limestone/dolomite bedrock surrounding and underlying all of the lower Great lakes. Arrow indicates study site In Northwest Lake Huron. Figure from Biddanda et al. 2012, published in *Nature Education Knowledge*, and originally sourced from Granneman et al. 2000 (Cited in the References). Note: Biddanda et al. 2012 is open access with permission to republish images therein elsewhere.

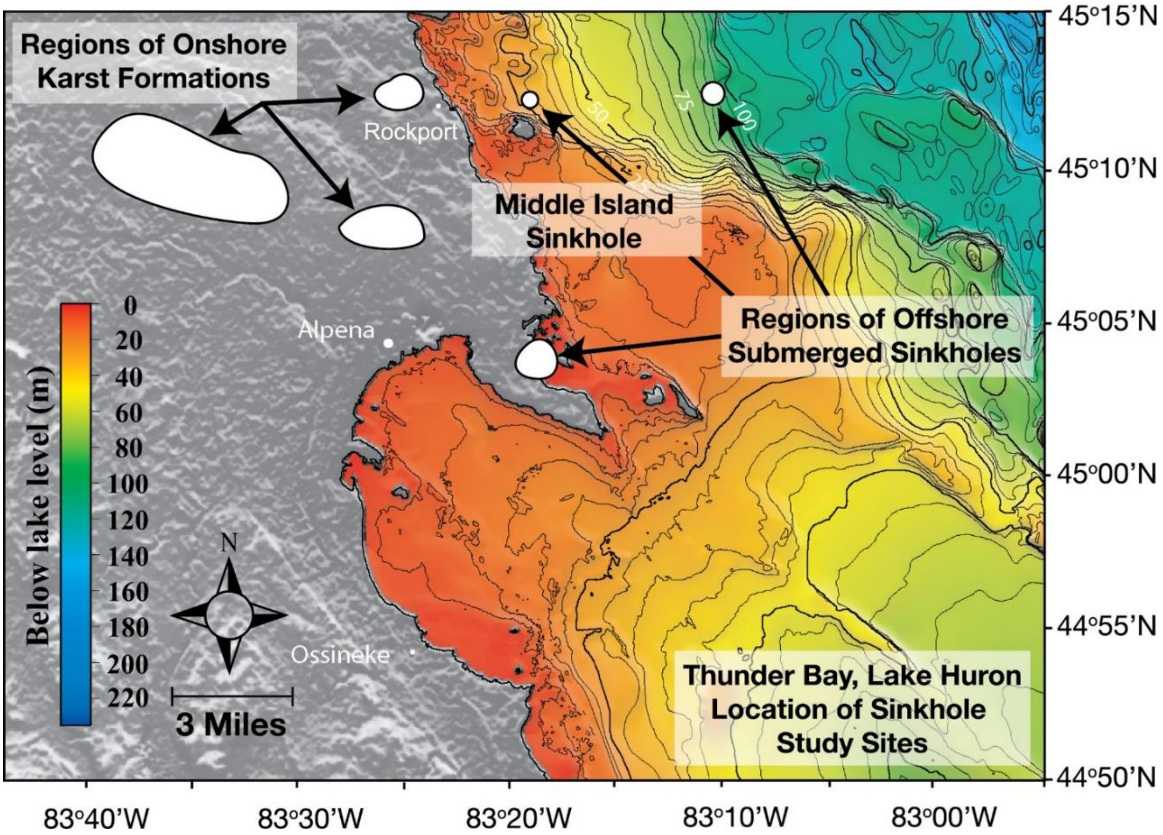

Figure S2. A bathymetric map of NW Lake Huron showing the location of nearshore and sunlit Middle Island Sinkhole and the offshore and aphotic Isolated Sinkhole. Lake Huron's submerged sinkholes: Known regions of on land karst formations in the northeast part of the lower peninsula of Michigan (USA), and underwater karst sinkholes in the Thunder Bay National Marine Sanctuary, Lake Huron (depth contours in 5m intervals). Site of the present study is the sunlit Middle Island Sinkhole at he 23 M isobath and the aphotic Isolated Sinkhole at the 110m isobath (directly East and offshore of Middle Island Sinkhole). Figure from Biddanda et al. 2012, published in *Nature Education Knowledge*, created by Cathy Darnell of NOAA-GLERL, and originally sourced from NOAA National Geophysical Data Center 1999 and Coleman 2002. Note: Biddanda et al. 2012 is open access with permission to republish images therein elsewhere.

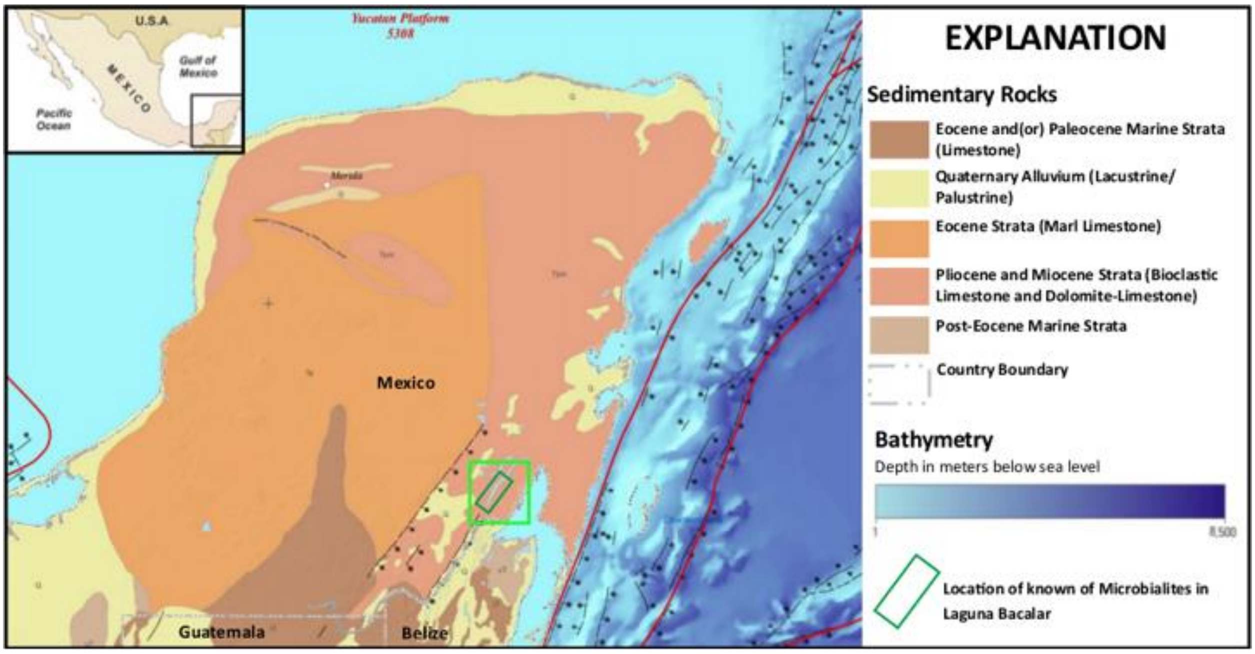

Figure S3. A map of the Yucatan Peninsula, Mexico, showing the geological context and the site where extensive reefs of microbialites occur in Laguna Bacalar. Geological map of the Yucatan peninsula, Mexico, showing the location of Laguna Bacalar (area covered by light green square inset) with extensive reefs of fringing microbialites (area enclosed by the dark green rectangle). Modified from Chrisopher et al. 2004 and Cabadas-Báes et al. 2018.

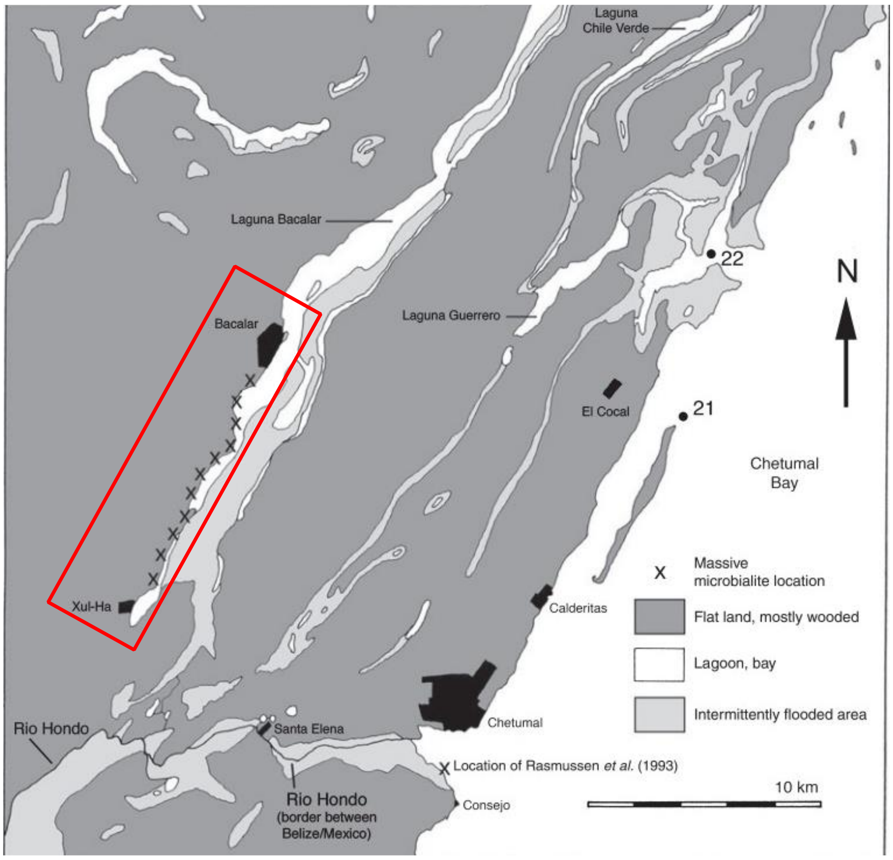

Figure S4. A map of Laguna Bacalar showing the location of known cenotes and microbialites that grow in and around the cenotes (sinkholes). Map of Laguna Bacalar showing known location of a series of cenotes (sinkholes, marked by crosses within the area marked by the red rectangle) and a continuum of massive microbialites occurring along the western shoreline of the southern half of of the lagoon in and around the cenotes constituting an extensive and contiguous living reef ~15km long (Modified from Gischler et al 2008 and Gischler et al. 2011).

Note: Each of these is accompanied by a fully explanatory legend.

References:

1. Biddanda, B.A.; Nold, S.C.; Dick, G.J.; Kendall, S.T.; Vail, J.H.; Ruberg, S.A.; Green, C.M. Rock, Water, Microbes: Sinkholes in Lake Huron are habitats for ancient microbial life. *Nat Ed Knowl* **2012**, 3 (10), 13. http://www.nature.com/scitable/knowledge/library/rock-water-microbes-underwater-sinkholes-in-lake-25851285
2. Cabadas-Báes, H.V.; Sedov, S.; Jiménez-Álvarez, S.P.; Leonard, D.; Lailson-Tinoco, B.; García-Moll, R.; Ancona-Aragón, I.I.; Hernández-Velázquez, M.L. Soils as a source of raw materials for ancient ceramic production in the Maya region of Mexico: Micromorphological insight. *Boletín de la Sociedad Geológica Mexicana* **2018**, 70 (1), 21–48, doi:10.18268/bsgm2018v70n1a2.
3. Christopher, D.; French, C.D.; Schenk, C.J. Map showing geology, oil and gas fields, and geologic provinces of the Caribbean Region: *U.S. Geological Survey Open-File Report 97–470K*, **2014**, doi.10.3133/ofr97470K.
4. Coleman, D.F. Underwater Archaeology in Thunder Bay National Marine Sanctuary, Lake Huron. *Mar Technol Soc J* **2002**, 36(3):33–44.
5. Gishler, E.; Gibson, M.A.; Oschmann, W. Giant Holoce Microbialites, Laguna Bacalar, Quintana Roo, Mexico. *Semimentology* **2008**, 55, 1293–1309, doi:10.1111/j.1365–3091.2007.00946.x.
6. Gishler, E.; Golubic, S.; Gibson, M.; Oschmann, W.; Hudson, J. Microbial mats and microbilaites of the freshwarter Lacuna Bacalar, Yucatan Peninsula, Mexico. In *Advances in Stromatolite Geobiology. Lecture Notes in Earth Sciences,* vol 131; Springer, Berlin, Heidelberg, **2011**, pp. 187–205, ISBN 978-3-642-10415-2.

7. Grannemann, N.G.; Hunt, R.J.; Nicholas, J.R.; Riley, T.E.; Winter, T.C. The importance of groundwater in the Great Lakes region. In: *USGS Water Resources Investigations Report 00-4008*, United States Geological Survey, Washington D.C., U.S.A, **2000**, pp. 1–13.
8. NOAA National Geophysical Data Center. Bathymetry of Lake Huron. *NOAA National Centers for Environmental Information* **1999**, doi.10.7289/V5G15XS5.
